# Direct transmission via households informs models of disease and intervention dynamics in cholera

**DOI:** 10.1371/journal.pone.0229837

**Published:** 2020-03-12

**Authors:** Victor A. Meszaros, Miles D. Miller-Dickson, Francis Baffour-Awuah, Salvador Almagro-Moreno, C. Brandon Ogbunugafor

**Affiliations:** 1 Department of Ecology and Evolutionary Biology, Brown University, Providence, RI, United States of America; 2 Department of Mathematics, Florida State University, Tallahassee, FL, United States of America; 3 Burnett School of Biomedical Sciences, University of Central Florida, Orlando, FL, United States of America; 4 National Center for Integrated Coastal Research, University of Central Florida, Orlando, FL, United States of America; Faculty of Science, Ain Shams University (ASU), EGYPT

## Abstract

While several basic properties of cholera outbreaks are common to most settings—the pathophysiology of the disease, the waterborne nature of transmission, and others—recent findings suggest that transmission within households may play a larger role in cholera outbreaks than previously appreciated. Important features of cholera outbreaks have long been effectively modeled with mathematical and computational approaches, but little is known about how variation in direct transmission via households may influence epidemic dynamics. In this study, we construct a mathematical model of cholera that incorporates transmission within and between households. We observe that variation in the magnitude of household transmission changes multiple features of disease dynamics, including the severity and duration of outbreaks. Strikingly, we observe that household transmission influences the effectiveness of possible public health interventions (e.g. water treatment, antibiotics, vaccines). We find that vaccine interventions are more effective than water treatment or antibiotic administration when direct household transmission is present. Summarizing, we position these results within the landscape of existing models of cholera, and speculate on its implications for epidemiology and public health.

## Introduction

Waterborne infections continue to pose a significant threat to human health. Among the most prominent of the waterborne infections is cholera, caused by the gram negative bacterium *Vibrio cholerae*, acquired through the consumption of contaminated water or food [1]. New epidemics are often driven by natural disasters or social conflict that facilitate the increased intake of contaminated water [2, 3]. As cholera is so strongly associated with the water supply, many public health intervention efforts have focused on the importance of water quality in preventing outbreaks. Recent studies have demonstrated that in certain settings, direct household transmission can serve an increasingly large role in disease in modern outbreaks [4–7]. We might especially expect direct household transmission to influence disease dynamics in high-density settings, like those associated with displaced populations (e.g. refugee camps) [8, 9].

The notion that cholera outbreaks can be driven or sustained by direct household transmission—which may occur between individuals within and between households—might alter the lens through which we study and potentially intervene in cholera outbreaks. Given how recently epidemiologists have engaged the specifics of direct household transmission, we have yet to fully appreciate the extent to which direct household transmission may alter the dynamics of cholera outbreaks. And while there are many important models of indirect cholera transmission via reservoirs [10–21], and some that examine the theoretical implications of direct household transmission [22, 23], we remain in the dark with regards to how recent insights on direct household transmission may specifically influence our picture of cholera disease dynamics and interventions.

In this study, we construct a mathematical model of cholera in a high-density setting, with random-mixing populations. Unlike prior studies, our model combines features of models that include waterborne transmission with parameters informed by recent empirical findings on direct household transmission. We observe that an outbreak in a high-density setting typified by a large amount of direct household transmission can resemble an outbreak driven exclusively by waterborne transmission, but with several critical differences, especially with regards to the effectiveness of interventions. Consequently, high-density cholera epidemics with direct household transmission suggest different intervention strategies in order to meaningfully suppress transmission. Our findings emphasize the need for more data on direct household transmission in high-density settings, as it can have important consequences on disease dynamics and optimal intervention measures.

## Model of disease dynamics with household transmission

### Description

We present a dynamical model of cholera based on a series of ordinary differential equations (ODEs). The model incorporates dynamics of both direct household transmission and disease flow through water reservoirs, as with other mathematical models of waterborne infections [22, 24]. We’ve codified our iteration as the Waterborne, Abiotic and other Indirectly Transmitted or (W.A.I.T.) framework, and will refer to it as such throughout this study.

Our model structure is adapted from a mathematical model of the 2010 outbreak in Haiti [10]. The model describes a cholera epidemic outbreak and is set with a population of 500000 (approximating the number of individuals in a mid-sized city in many countries affected by cholera).

In order to interrogate the under-examined role of direct household transmission within a cholera epidemic, the model allows us to investigate the impact of direct transmission of the susceptible population as a proxy for transmission within and between households. Throughout the text, we refer to all non-waterborne, environmental transmission in this model as “direct,” “household,” or “direct household” transmission. We use the terms interchangeably. By constructing a framework which allows us to combine different routes of transmission, we are afforded the opportunity to observe how disease burden and ideal intervention strategies are influenced by different transmission strategies.

$$\frac{dS}{dt}=\pi N+\omega R+\epsilon V-\mu S-\alpha S\frac{W_{L}}{\kappa_{L}+W_{L}}-\alpha S\frac{W_{H}}{\kappa_{H}+W_{H}}-\tau S-\eta (I+A)S$$ (1)

$$\begin{array}{cc} \frac{dI}{dt} & =\left(1-p\right)\alpha S\frac{W_{L}}{\kappa_{L}+W_{L}}+\left(1-p\right)\alpha S\frac{W_{H}}{\kappa_{H}+W_{H}}\\ & \;-(\mu_{c}+\mu +\left(1-\theta \right)\gamma +\theta \gamma \lambda )I+\left(1-p\right)\eta S\left(I+A\right) \end{array}$$ (2)

$$\frac{dA}{dt}=p\alpha S\frac{W_{L}}{\kappa_{L}+W_{L}}+p\alpha S\frac{W_{H}}{\kappa_{H}+W_{H}}-\left(\mu +\gamma \right)A+p\eta S\left(I+A\right)$$ (3)

$$\frac{dR}{dt}=((1-\theta )\gamma +\theta \gamma \lambda )I+\gamma A-(\mu +\omega )R$$ (4)

$$\frac{dV}{dt}=\tau S-(\epsilon +\mu )V$$ (5)

$$\frac{dW_{H}}{dt}=\left(\psi \theta +\left(1-\theta \right)\right)\frac{\xi_{s}}{W}I+\frac{\xi_{A}}{W}A-\chi W_{H}$$ (6)

$$\frac{dW_{L}}{dt}=\chi W_{H}-\delta W_{L}$$ (7)

### The compartmental diagram

**Fig 1:**
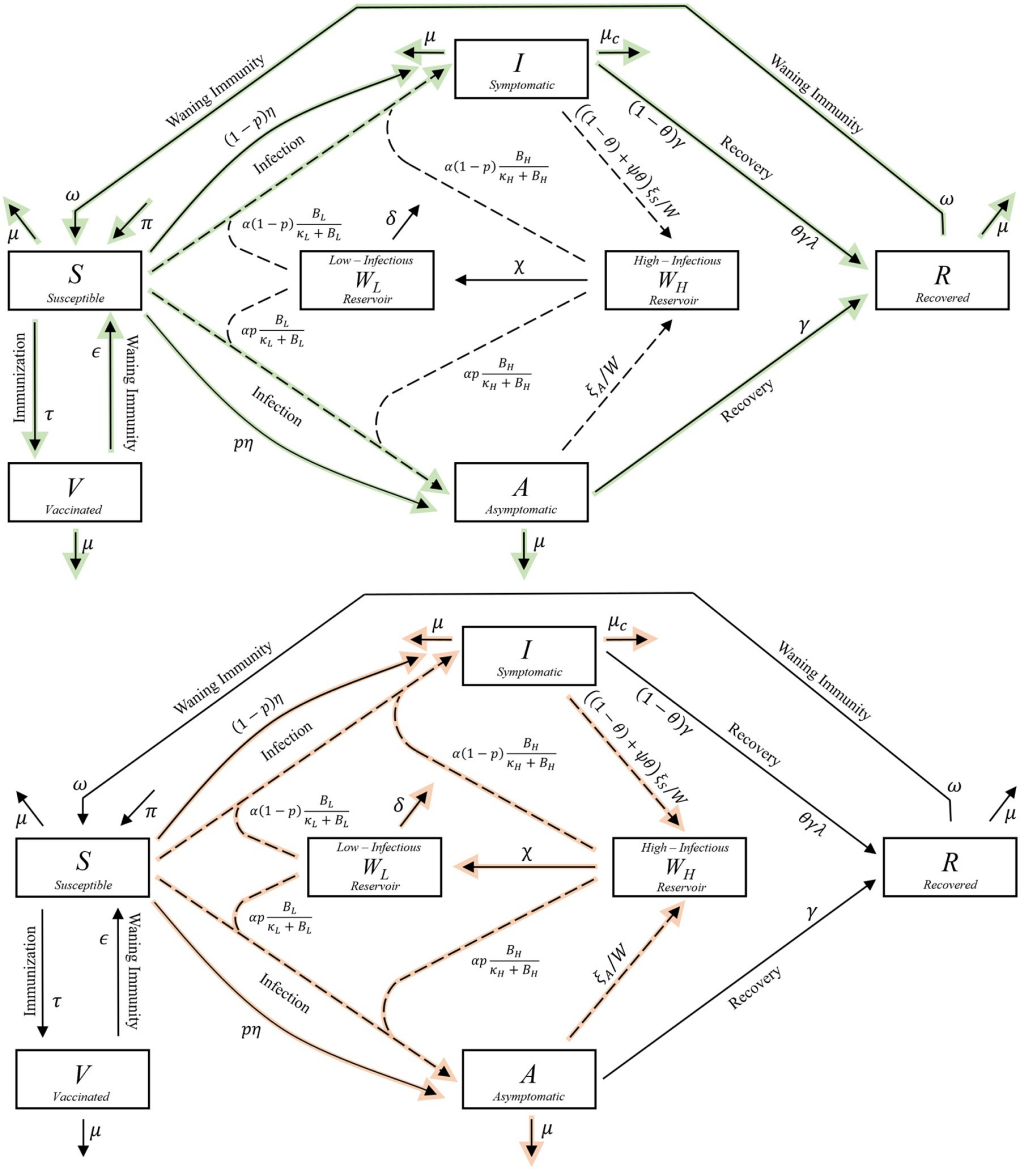
A Waterborne, Abiotic and other Indirectly Transmitted (W.A.I.T.) dynamical compartmental model of cholera. **Top**: This diagram is a variation of a standard compartmental diagram. Green arrows highlight the flow of the population of hosts through the system. **Bottom**: here the red arrows highlight flow of disease through the system.

The model can be visualized using a modified compartmental diagram that contains information on the spread of disease agents and hosts through the system, as seen in Fig 1. The model features seven ordinary differential equations—one for each compartment—and twenty-two parameters. The compartments *S*, *I*, *A*, *R* and *V* represent populations of susceptible, symptomatically infected, asymptomatically infected, recovered and vaccinated individuals respectively. The *W*_*H*_ and *W*_*L*_ compartments represent total *V. cholerae* bacteria, of each type (high or low infectious) within a single water reservoir accessible to the sum total of individuals within the population.

Some model features, which have also reflected findings in prior studies [10, 25, 26], include the following:

- Disease is acquired by susceptible individuals *S*, via infected drinking water from either of the two reservoirs, or through direct household transmission. The fraction of *S*-individuals that move either to *A* or *I* is then given by *p* and 1 − *p* respectively
- The reservoir compartments *W*_*H*_ and *W*_*L*_ differ in how much *V. cholerae* is required from each to infect an individual, with the high-infectious reservoir (*W*_*H*_) having a lower minimum infectious dose. With time, populations of *V. cholerae* within the high-infectious reservoir naturally decay to a population with lower infectiousness.
- There are two groups of infected individuals: *I*-infected individuals have significantly higher excretion rates and can be treated with antibiotics, decreasing their excretion rate. *A*-infected individuals have lower natural excretion rates.
- Individuals enter and exit the system via a constant natural birth rate as well as infected and uninfected death rates.
- Infection is removed from the system either via bacterial decay in the low-infectious reservoir, or via the death of infected individuals.
- The number of *S*-individuals that are vaccinated is modeled as a fraction of the total population size, and individuals return to the susceptible compartment as their vaccine induced immunity wanes.
- *I* and *A*-individuals recover from the disease at a natural rate, *I*-individuals treated with antibiotics recover at a faster rate, and the recovered state provides a temporary immunity which, after waning, will feed individuals back into the susceptible population.
- Importantly, the direct transmission parameters are based on an empirical study of household transmission in Bangladesh [7].

### Analytic equations and parameters

The set of ordinary differential equations (Eqs 1–7) define the dynamics of the system. Direct household transmission is implemented through the term *η*. This term translates to the total daily fraction of all possible interactions between susceptible individuals and infected individuals (symptomatic and asymptomatic) that lead to an infection. As stated previously, household transmission is implemented using an empirical study that estimated rates of transmission of cholera via households in Bangladesh [7].

In our modeling scheme (W.A.I.T.), environmental dynamics are also realized within their own set of differential equations. The $\alpha S\frac{W_{L,H}}{\kappa_{L,H}+W_{L,H}}$ terms quantify the likelihood that individuals become infected with *V. cholerae* given a daily water consumption rate *α* and a minimum infectious dose *κ* for each reservoir.

For either route of transmission (household or waterborne) the parameter *p* scales the rate of transmission by serving as the fraction of individuals that move to the *A*-compartment (asymptomatic), whereas 1 − *p* serves as the fraction that move to the *I*-compartment (symptomatic). *p* is, therefore, a population-level expression of host factors.

Other notable terms include *τ*, the vaccination rate, which represents the daily fraction of *S*-individuals that receive vaccinations; *ξ*_*s*_, the symptomatic individuals excretion rate, which is three orders of magnitude larger than that of the asymptomatic case and can be reduced via antibiotic administration; λ, the relative recovery rate of those receiving antibiotics; and *θ*, the proportion of symptomatic individuals receiving antibiotics. Table 1 lists the parameters and values used in this simulation.

**Table 1:** Cholera household (direct) transmission model parameters.

Label	Value	Units	Definition	Sources
*α*	0.002	(*person* ⋅ *day*)^−1^	Rate of water consumption	[29]
*L*	10^5^	*cells*/*day*	Low-infectious *V.cholerae*	[30]
*H*	2000	*cells*/*day*	High-infectious *V.cholerae*	[31]
*χ*	1	%/*day*	Rate of decay in high-infectious reservoir	[32]
*δ*	$0.3\bar{3}$	%/*day*	Environmental death rate of *V. cholerae*	[25]
*μ*	4.49 ⋅ 10^−5^	%/*day*	Natural birth & death rate	[33]
*μ*_*c*_	0.046	#/*day*	Symptomatic mortality rate	[34]
*π*	5.48 ⋅ 10^−5^	#/*day*	Fixed fractional birth rate	[34]
*γ*	0.20	%/*day*	Disease recovery rate	[35]
*p*	0.79	%/*day*	Proportion of asymptomatic cases	[36]
*ξ*_*S*_	1.30 ⋅ 10^11^	*cells*/*day*	Symptomatic excretion rate	[37]
*ξ*_*A*_	1.30 ⋅ 10^8^	*cells*/*day*	Asymptomatic excretion rate	[38]
*ω*	3.42 ⋅ 10^−3^	%/*day*	Waning of natural immunity	[39]
*τ*	0.25	%/*day*	Vaccination rate	[40]
*ϵ*	1.37 ⋅ 10^−3^	%/*day*	Waning vaccine induced immunity	[41]
*θ*	0.10	%/*day*	Symptomatic persons receiving antibiotics	[42]
*ψ*	0.52	%/*day*	Shedding rate of antibiotic treated persons	[43]
λ	2.3	%/*day*	Relative recovery rate, receiving antibiotics	[35]
*η*	5^−8^	%/*day*	Total daily average infectious direct interactions	[7]
*W*	1.5	*Deciliters*	Size of water reservoir	[44]
*N*	5 ⋅ 10^5^	*People*	Number of individuals	—

The initial conditions used across all simulations included a population of 500 000 susceptible individuals with a small initial amount of *Vibrio cholerae* present in each reservoir to prime the outbreak (5 cells at *t* = 0). Additionally, the values of the symptomatic, asymptomatic, vaccinated, and recovered compartments were set to zero at the start of each run.

### Parameter sensitivity analysis and basic reproductive ratio

The basic reproductive number, *R*_0_ is a contentious measure in epidemiology, but can be interpreted as a signature for the average infectiousness of a pathogen in a given setting [27, 28]. In this model, given the nominal parameters, we find that **R**_**0**_ = **8.37**, this value is in agreement with reproductive ratio ranges (*R*_0_ = 3–19) determined from past epidemics and computational models of cholera [19, 25]. Estimates for the reproductive ratio of cholera vary within the literature, which correspond to different epidemic settings and contexts. In the S1 File, we outline how the numerical value for *R*_0_ was computed in the model presented in this study.

**Fig 2:**
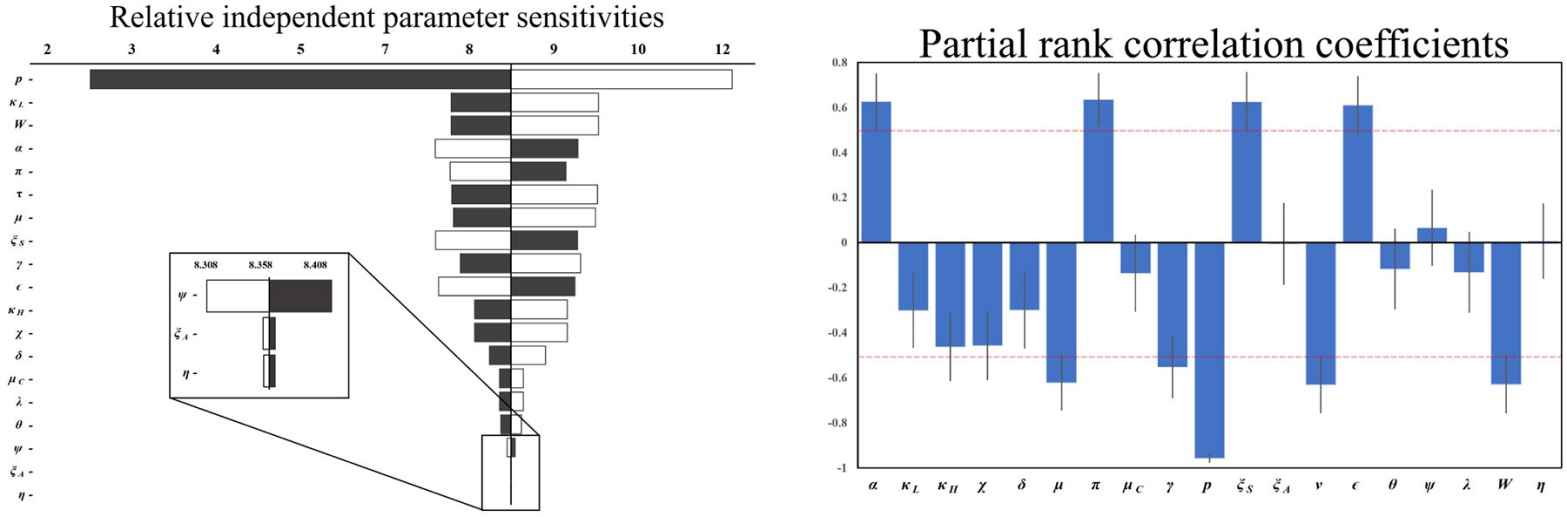
R_0_ sensitivity analysis. **Left**: A tornado plot showing the independent sensitivity of *R*_0_ to changes in the model parameters. Black bars indicate the value of *R*_0_ when the associated parameter is *increased* by 25% from its nominal value. White bars indicate the value of *R*_0_ when the associated parameter is *decreased* by 25%. **Right**: A Partial Rank Correlation Coefficient (PRCC) analysis was preformed with respect to *R*_0_ highlighting the inter-correlated sensitivities of each of the model parameters. The blue bars show the mean value of each PRCC, with error bars at one standard deviation. This analysis was preformed by sampling over uniform distributions of 15% around the nominal model parameter values in line with the methods provided by Blower and Dowlatabadi (1994) [45].

The relative sensitivity of *R*_0_ to changes in the model parameters was examined. Both an independent parameter sensitivity analysis as well as a partial rank correlation coefficient (PRCC) analysis [45] were conducted. The PRCC was performed at 500 iterations with 50 samples per iteration. Fig 2 shows the results of these investigations. Here we find that *p*, *κ*_*L*_, *W* and *α* have the greatest independent (uncorrelated) sensitivity, while *p*, *π*, *W*, and *α* hold the greatest interrelated sensitivity by mean value. These parameters include two parameters strongly related to environmental aspects of the model: the rate of water consumption *α*, and the total water reservoir size *W*. Both the independent and the correlation sensitivity analyses are in agreement with regards to the sensitivity of the model parameters on the *R*_0_.

Of note, *p* (proportion of asymptomatic cases) is the most sensitive parameter within the model by a large margin. The fraction of infected individuals that become asymptomatic can be considered as an average characteristic of the model population. By our assessment, the high sensitivity in *R*_0_ due to *p* is caused by the relatively large distinction between the excretion rates of symptomatic (*ξ*_*S*_) and asymptomatic (*ξ*_*A*_) individuals (*ξ*_*S*_/*ξ*_*A*_ ∼ 10^3^). Given this, if the fraction of individuals that move into the asymptomatic compartment, *p*, is increased, the basic reproductive ratio is noticeably reduced.

At the disease-free equilibrium—the regime where *R*_0_ is calculated—the direct transmission term *η* shows low sensitivity to the basic reproductive number, indicating that variations in the household transmission rate may not lead to significant variations in the *R*_0_ value. Thus, whereas the rate *η* may have a noticeable impact on the dynamical peak of the infection, or on the time scales associated with the outbreak of the infection, it may not largely impact how explosive (in terms of *R*_0_) the contagion is initially. More generally, this can be interpreted as a limitation of the *R*_0_ metric with regards to the epidemic features that it captures: it is not all-encompassing. In addition to *R*_0_, a full and proper understanding of the epidemic dynamics requires a thorough analysis of model-specific features (e.g. the behavior of the peak of the infection).

**Fig 3:**
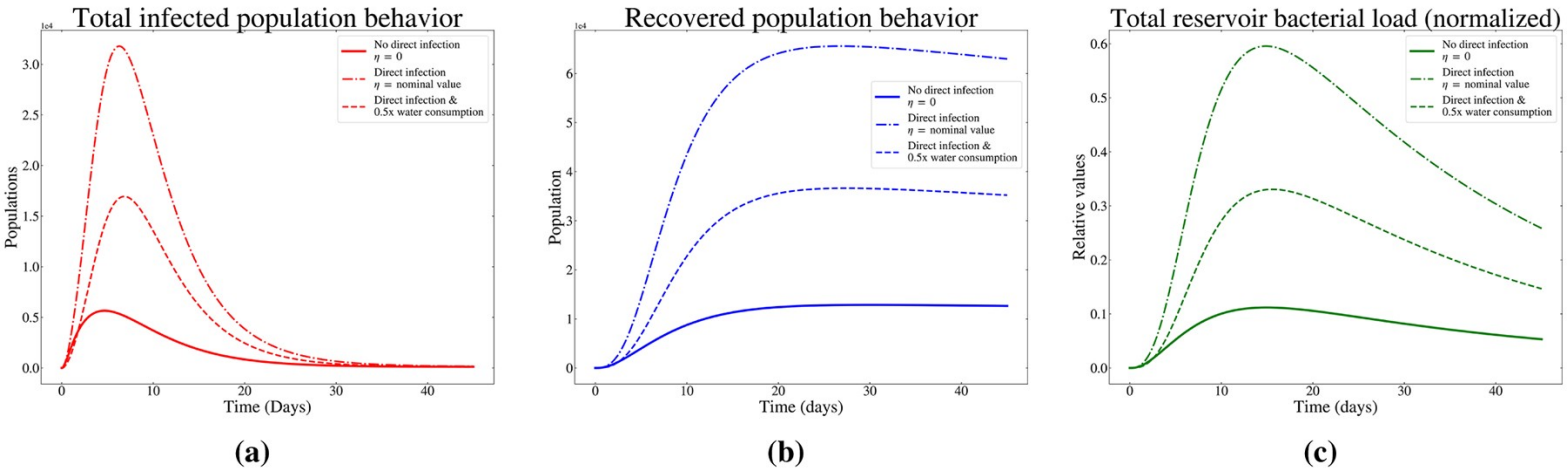
Basic model dynamics as a function of household transmission and water treatment: Of the (a) infected and (b) recovered populations, as well as the (c) combined bacterial load of the water reservoirs, normalized to maximum bacterial load value across all 3 cases. Lines correspond to different epidemic conditions, including a no household transmission scenario, one with household transmission and one where household transmission is coupled with a water treatment intervention (which decreases the amount of transmission through consumption of contaminated water).

The infected populations in our model (both asymptomatic and symptomatic) initially increase—moving away from the disease-free equilibrium—and eventually reach stable non-zero (although small) equilibrium values (see Fig 3(a)). This is consistent with *R*_0_ > 1, as well as the eigenvalue analysis of the Jacobian matrix at the disease-free equilibrium (shown in the Supplemental Appendix). The eigenvalues of the Jacobian matrix for the ODE system at the disease-free equilibrium are all real numbers, with two values greater than zero, indicating that the disease-free equilibrium is unstable. We present the Jacobian matrix of the system and its corresponding eigenvalues in the Supplemental Appendix.

### Model dynamics: The added effects of direct household transmission

**Fig 4:**
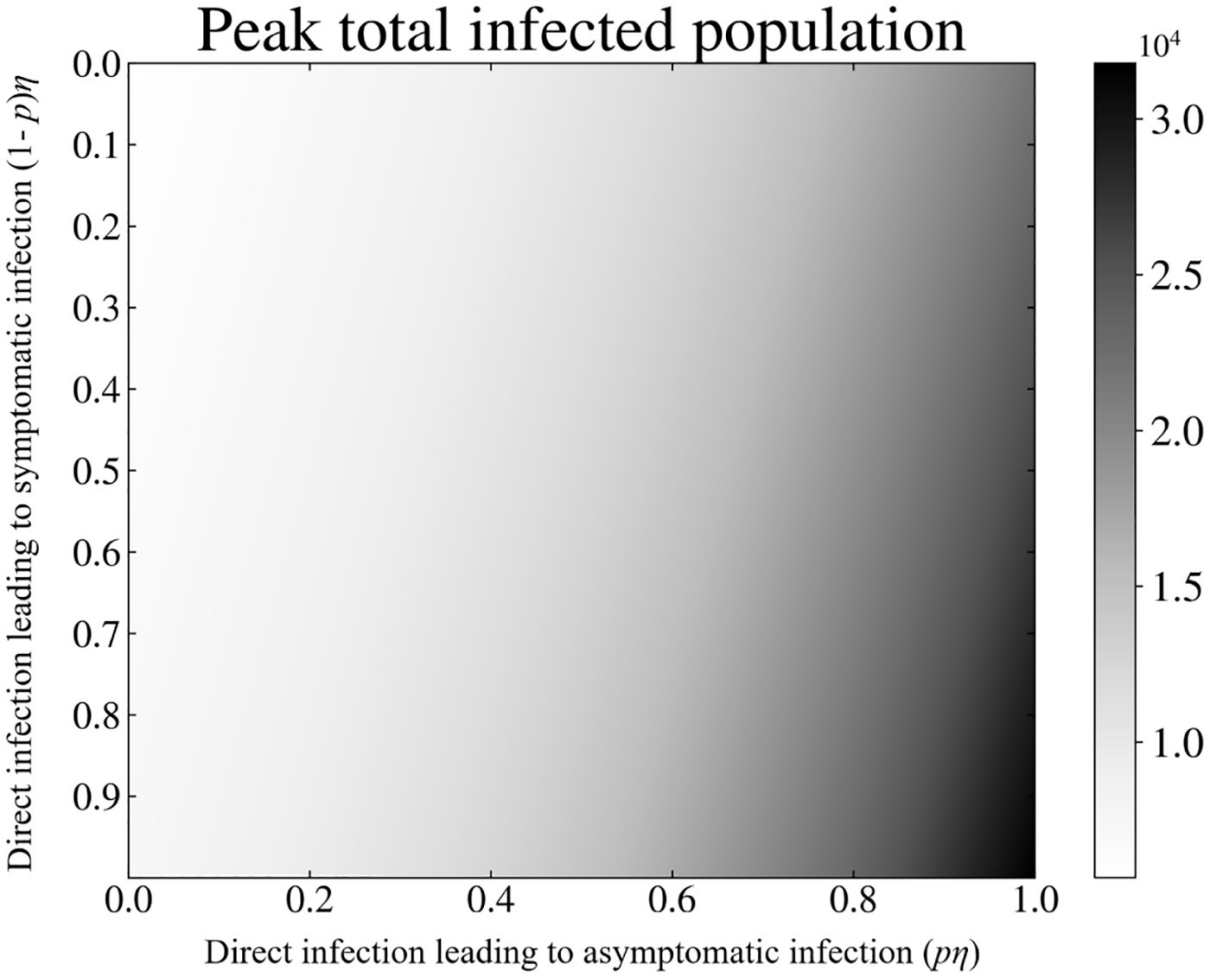
Peak infected population behavior: A heat map of peak total infected population as a function of household (direct) transmission terms (1 − *p*)*η* and *pη*. Along the X and Y axis, the household transmission terms are notated as multiples of their nominal model values as seen in Table 1. The top left corner denotes the region where *pη* = (1 − *p*)*η* = 0, and no household transmission occurs. While the bottom right corner denotes the region where *η* = nominal model value. The gray-scale intensity within the heat map represents the peak total infected population. Comparing the intensity of the top left corner to the bottom right we see an increase in the peak total infected population as household transmission is introduced and its effects increased.

**Fig 5:**
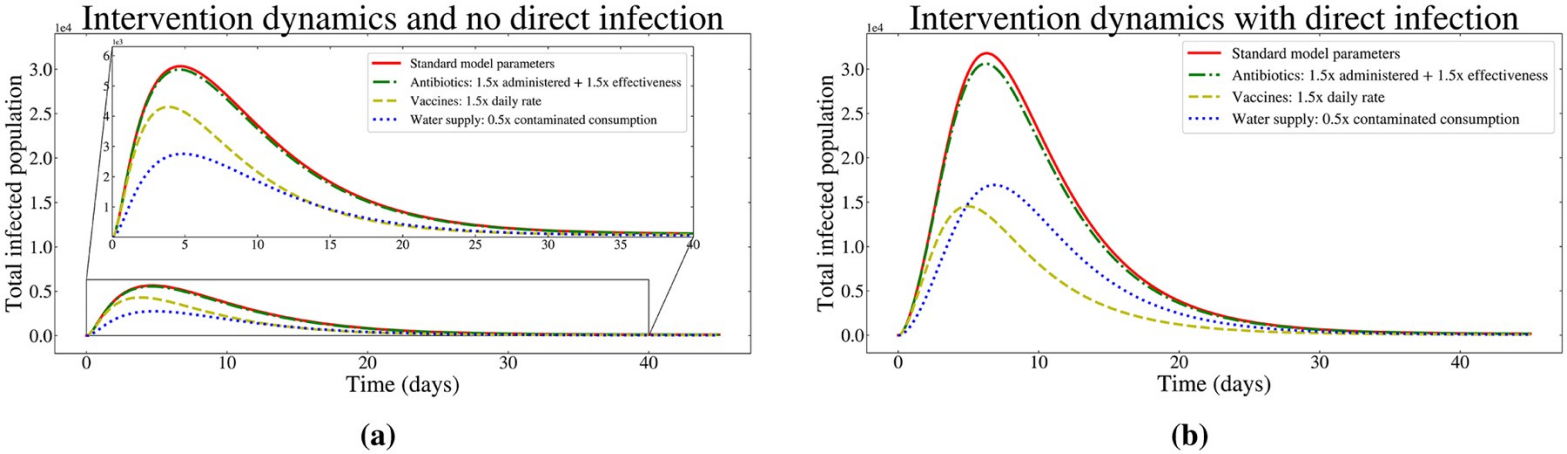
Comparing the effectiveness of interventions in the setting of household transmission. There are three hypothetical intervention scenarios, and their effect on the dynamics of the total population of infected individuals in the cases when household (direct) transmission is (a) absent and (b) present. The primary Y-axes of fig(a) and (b) are identical. The inset in fig(a) highlights the regions, *x* = 0 to 40 (days) and *y* = 0 to 6 ⋅ 10^3^ in order to better distinguish the differences in the dynamics of the curves.

Introduction of infection between individuals within and between households into models of cholera has a substantial impact on the dynamics of outbreaks. In order to showcase this impact, three scenarios were investigated with simulations: (i) No household transmission (*η* = 0), (ii) household transmission with standard water consumption (*η* & *α* = nominal values), and (iii) household transmission with water treatment (decreased consumption of contaminated water) (*η* = nominal value and 0.5*α*). Simulations were run at a time resolution of 10,000 timesteps over a period of 60 days for all three cases. Figs 3–5 depict various aspects of the dynamics of disease as well as the effects of potential interventions in each scenario. Fig 3(a) demonstrates a 5.63x increase in the peak number of total infected individuals when realistic household transmission rates are introduced [7]. Fig 3(b) shows the increased peak values of the total number of recovering individuals, as more agents are drawn into the recovered compartments due to the increase in the population of infected individuals. This correlation is an example of how household transmission increases the intensity of the epidemic within the model. The total flow of individuals moving through the system increases in correspondence with an increase in household transmission. In the Supplemental Appendix, we provide information on how the different transmission scenarios influence the total number of infections across the duration of the model.

Fig 4 highlights properties of the peak total infected population, and how it is modified by household transmission. While there are several features of an epidemic that one could use to capture this influence, we consider the peak number of infected individuals because it signifies the state where the infection is exacting its greatest toll. Each of the 250 000 grey-scale pixels that compose the heat-map represents a single run of the cholera household transmission model. For each run, we plot the peak of the total (combined *I* + *A*) infected population, modulating the rate of transmission into the *A* and *I* compartments via the direct transmission terms, *pη* and (1 − *p*)*η* respectively. All other model parameters are kept constant, held at their nominal values as shown in Table 1. The x and y axes show fractions of the nominal values of *pη* and (1 − *p*)*η*, respectively, from Table 1. At the top left corner where *pη* = (1 − *p*)*η* = 0, there is no household transmission present. The peak total infected population here is at its lowest value, 5647. This is equal to the same value we see at the peak of the solid curve in Fig 3(a). As we increase *η* and thus the amount of household transmission present, we see a dramatic increase in the peak number of total infected individuals (lower right corner).

The largest peak value that we observe, 31787, appears in the lower right corner of the plot where the household transmission terms are their largest. This value corresponds to the peak of the dashed-dotted line in Fig 3(a). Implementing direct household transmission in this model thus increases the peak total infected population by approximately 5.63x. Of note: *p* is expected to be an approximately fixed property of hosts, whereas direct household transmission is modulated solely by the *η*-term, representing varying levels of inter- and intra-household transmission. Thus, we only consider the peak infection values along the diagonal elements of the heat map as representing relevant (or realistic) scenarios. The rest of the space on the heat map showcases the extent to which host-level affinities for symptomatic or asymptomatic infection-types can affect the peak levels of infection.

Like several other models, the W.A.I.T. modeling scheme utilized in this study allows us to independently model the dynamics of the environmental reservoirs via a set of separate differential equations. In Fig 3(c), we observe that the normalized total reservoir bacterial load peak is 2.95x larger for the case where household transmission is present. Additionally, the peak bacterial load is shifted 0.63x of a day later in the simulation when household transmission is introduced. This “late arrival” phenomenon is also observed, within the infected and recovered populations. This indicates, within the scope of the model, that the overall duration of the epidemic is also slightly increased when household transmission is introduced.

### Intervention schemes

Having explored the impact of household transmission on disease dynamics, we then analyzed a range of potential sample interventions: vaccination, antibiotic administration, and water treatment (decreasing contaminated water consumption). Each were realized by modifying model parameters relevant to that intervention. We compare the impact of these interventions in cases with and without household transmission. Note that these intervention schemes assume implementation occurs at the onset of the infection.

Fig 5 depicts the total number of both symptomatic and asymptomatic infected individuals in settings utilizing various intervention schemes. In the case where no direct household transmission is present (Fig 5a), decreasing the consumption of contaminated water (*α*) has a strong effect on disease dynamics: a 0.5x decrease in contaminated water consumption provides an about 0.5x decrease in the peak number of infected individuals. In a similar setting with no direct transmission, a 1.5x increase in vaccination and antibiotic interventions provide a smaller relative decrease in the peak number of infected individuals. This demonstrates that for cholera epidemics driven largely by the waterborne transmission, and not through household transmission, one would expect (unsurprisingly) that targeting the water consumption would be the best way to attenuate the epidemic.

Alternatively, our explorations of settings featuring high levels of household transmission (Fig 5b) show that vaccine interventions emerge as the most effective strategy. In settings featuring high levels of household transmission, we predict that emergency vaccination programs would be more effective than water treatment-based interventions. The reasoning behind this: vaccination directly modulates the fraction of person-to-person contacts that involve an infected individual by removing infected individuals from the population, and preventing an explosive outbreak from getting going. In settings with little household transmission, vaccination indirectly mitigates the spread of infection by removing infected individuals that could contribute to the transmission cycle by infecting the water reservoir.

## Discussion

In this study, we propose a modeling framework for cholera in high-density, well-mixed population settings that fully encompasses two different routes of transmission: standard transmission via an environmental reservoir intermediate (waterborne), and directly between hosts in and between households. The question of how these two routes interact is important on the backdrop of relatively new findings that highlight the importance of household transmission [4–7]. Importantly, our model structure allows one to examine how different magnitudes of household transmission influence disease dynamics. A recent model of high-density settings simulated these dynamics effectively, but did so for a single refugee camp with a specific set of parameter values [9]. Our model, alternatively, explores the space of possible direct transmission parameters that may pertain to different high-density cholera settings.

We find that variation in household transmission changes how the disease is spread. Interestingly, the presence of direct transmission not only influences *R*_0_, but rather, it changes specific features of disease that cannot be captured by the *R*_0_. For example, variation in household transmission influences both the peak number of cases in an outbreak and the duration of a given outbreak. Whereas, the *R*_0_ speaks more directly to the “explosiveness” of an outbreak, how rapidly it progresses initially. This result has relevance for understanding specifics of cholera outbreaks and for general canon in mathematical epidemiology. With regards to cholera outbreaks, household transmission can perniciously influence disease outcomes by extending the severity and duration of a given outbreak. With regards to broader conversations in mathematical epidemiology, the household transmission case highlights the limits of *R*_0_ as a tool for assessing the severity of epidemics. While it remains a powerful proxy for certain characteristics of an outbreak, undue focus on it as the defining characteristic of an epidemic can be misleading.

As cholera remains a major global health problem in high-density, temporary settlements, our results suggest that the public health sector should be mindful of how sensitive outbreak dynamics are to different transmission characteristics. Specifically, the nature and amount of household transmission can have a meaningful impact, not only on the trajectory of disease, but on which intervention strategies might be most effective in preventing or attenuating a given epidemic. Our results support recent findings suggesting that vaccines can be especially effective in high-density settings with large amounts of household transmission [9]. That is, water treatment and antibiotics—both of which may be relevant in other settings—are likely to be less effective in subverting epidemics in refugee camps, and related settings where household transmission is high. One can explain this by highlighting the nature of high-density settings: transmission can occur too quickly (between individuals) for more structural-based (e.g. water treatment) or post-infection (e.g. antibiotics) interventions to have as meaningful an effect. Vaccines, however, can lower the peak of infection by decreasing the number of individuals entering a high-density setting, preventing both the number of individuals capable of direct transmission and the contribution of disease into the common water supply.

In closing, we urge epidemiologists to appreciate that epidemics are diverse ecological phenomena, where different settings may require peculiar and specific interventions. Our findings implore the research community to study epidemics with an increased level of granularity, as essential properties can be defined by particulars.

## Supporting information

S1 File Supplemental appendix. Here we describe a set of additional analyses and calculations relevant to content in the main text. These include:
Calculation of the sum total of cases as a function of direct infectionAn outline of the method for determining the reproductive ratio (*R*_0_) for the Cholera household (direct) infection model.Discussion of the stability of the system at the disease free equilibrium.
(PDF)
