# Supplementary material for: Direct transmission via households informs models of disease and intervention dynamics in cholera
Source: PLoS One. 2020 Mar 12;15(3):e0229837. doi: 10.1371/journal.pone.0229837 (PMC7067450; doi:10.1371/journal.pone.0229837)
Supplement: S1 File — Here we describe a set of additional analyses and calculations relevant to content in the main text. These include: Calculation of the sum total of cases as a function of direct infectionAn outline of the method for determining the reproductive ratio (R0) for the Cholera household (direct) infection model.Discussion of the stability of the system at the disease free equilibrium. (PDF) [file pone.0229837.s001.pdf]

S1 File. Supplemental Appendix

Direct transmission via households informs models of disease and intervention dynamics in cholera

Victor A. Meszaros[1,◑], Miles D. Miller-Dickson[1,◑], Francis Baffour-Awuah Junior[2], Salvador Almagro-Moreno[3,4], C. Brandon Ogbunugafor[1,*]

1 Department of Ecology and Evolutionary Biology – Brown University
80 Waterman Street, Providence RI, 02912 USA
2 Department of Mathematics – Florida State University
1017 Academic Way, Tallahassee, FL, 32304 USA
3 Burnett School of Biomedical Sciences – University of Central Florida
6850 Lake Nona Boulevard, Orlando, FL, 32827 USA
4 National Center for Integrated Coastal Research – University of Central Florida
4000 Central Florida Boulevard, Orlando, FL, 32816 USA

◑ These authors contributed equally to this work.
* brandon_ogbunu@brown.edu

Below we describe a set of additional analyses and calculations relevant to content in the main text. These include:

- Calculation of the sum total of cases as a function of direct infection
- An outline of the method for determining the reproductive ratio (R_0) for the Cholera household (direct) infection model.
- Discussion of the stability of the system at the disease free equilibrium

Model dynamics: sum total values

In S1 Table we present the summed total number of counts for all nine dynamic cases considered in Fig 3. These values can be thought of analogous to the area under each of the nine curves in Fig 3a-c. Of note, given that the three curves in fig 3c are normalized to the maximum amount of Cholera summed across both the high and low infectious reservoirs; the values reported in column 3 of S1 Table are that of the sum total amount of bacteria across both reservoirs for each of the three model runs.

Basic reproductive ratio – $\mathbf{R_0}$

The numeric value for the basic reproductive ratio R_0 of *V. cholera* referenced in the main text, and used as part of our sensitivity analysis, was derived following the methods of Diekmann et. al. 2009 [1].

	**Total infected cases**	**Total recovered cases**	**Total bacterial count** ($W_H + W_L$)
**No direct infection** $\eta = \mathbf{0}$	$1.55 \cdot 10^{7}$	$1.04 \cdot 10^{7}$	$8.99 \cdot 10^{11}$
**direct infection** $\eta =$ **nominal value**	$7.74 \cdot 10^{7}$	$5.24 \cdot 10^{7}$	$4.53 \cdot 10^{12}$
**direct infection &** $0.5x$ **water consumption**	$3.20 \cdot 10^{7}$	$2.88 \cdot 10^{7}$	$2.51 \cdot 10^{12}$

S1 Table. In S1 Table we present the summed total number of counts for all nine dynamic cases of this model considered in Fig 3. Of note, the values reported in column 3 of S1 Table are that of the sum total amount of bacteria across both reservoirs for each of the three model runs.

Firstly, we construct the arrays $t = (t_0, t_1, ..., t_m)$ and $\sigma = (\sigma_0, \sigma_1, ..., \sigma_m)$. The ith element of t, denoted t_i, is defined to be the rate term associated with the flow of *new* infection *into* the ith compartment. That is, the flow of infection between two infected compartments is not included in t. The ith element of σ, denoted σ_i, is defined to be the sum of all other rate terms associated with flows into or out of the ith compartment. That is, the total rate of change of the ith compartment is given by $t_i + \sigma_i$. In calculating R_0, it suffices to restrict the index i in t_i and σ_i to infected compartments only. In the case of *V. cholerae*, these are the compartments I, A, W_L, and W_H. Below we present the elements of t and σ at the disease free equilibrium.

$$t = \begin{pmatrix} \frac{\pi(A+I)\eta N(1-p)(\mu+\epsilon)}{\mu(\mu+\tau+\epsilon)} + \frac{\pi\alpha N(1-p)W_H(\mu+\epsilon)}{\mu\kappa_H(\mu+\tau+\epsilon)} + \frac{\pi\alpha N(1-p)W_L(\mu+\epsilon)}{\mu\kappa_L(\mu+\tau+\epsilon)} \\ \frac{\pi\eta Np(A+I)(\mu+\epsilon)}{\mu(\mu+\tau+\epsilon)} + \frac{\pi\alpha NpW_H(\mu+\epsilon)}{\mu\kappa_H(\mu+\tau+\epsilon)} + \frac{\pi\alpha NpW_L(\mu+\epsilon)}{\mu\kappa_L(\mu+\tau+\epsilon)} \\ 0 \\ \frac{A\xi_A}{W} + \frac{I(\theta\psi-\theta+1)\xi_S}{W} \end{pmatrix} \qquad \sigma = \begin{pmatrix} I\left(\gamma\theta\lambda + \gamma(1-\theta) + \mu_c + \mu\right) \\ A(\gamma+\mu) \\ -\chi W_H + \delta W_L \\ \chi W_H \end{pmatrix}$$

We calculate the corresponding T and Σ matrices. These are $m \times m$ matrices (in this case $m = 4$) defined by,

$$T_{ij} = \frac{\partial t_i}{\partial X_j}(x_0)$$
$$\Sigma_{ij} = \frac{\partial \sigma_i}{\partial X_j}(x_0)$$

where X_j is the jth agent, selected from the m agents associated with t and σ, and x_0 is the disease-free equilibrium of the model. Calculating these derivatives for the *V. cholera* model one finds,

$$
T = \begin{pmatrix} \frac{\pi\eta N(1-p)(\mu+\epsilon)}{\mu(\mu+\tau+\epsilon)} & \frac{\pi\eta N(1-p)(\mu+\epsilon)}{\mu(\mu+\tau+\epsilon)} & \frac{\pi\alpha N(1-p)(\mu+\epsilon)}{\mu\kappa_L(\mu+\tau+\epsilon)} & \frac{\pi\alpha N(1-p)(\mu+\epsilon)}{\mu\kappa_H(\mu+\tau+\epsilon)} \\ \frac{\pi\eta Np(\mu+\epsilon)}{\mu(\mu+\tau+\epsilon)} & \frac{\pi\eta Np(\mu+\epsilon)}{\mu(\mu+\tau+\epsilon)} & \frac{\pi\alpha Np(\mu+\epsilon)}{\mu\kappa_L(\mu+\tau+\epsilon)} & \frac{\pi\alpha Np(\mu+\epsilon)}{\mu\kappa_H(\mu+\tau+\epsilon)} \\ 0 & 0 & 0 & 0 \\ \frac{(\theta\psi-\theta+1)\xi_S}{W} & \frac{\xi_A}{W} & 0 & 0 \end{pmatrix}
$$

$$
\Sigma = \begin{pmatrix} \gamma\theta\lambda + \gamma(1-\theta) + \mu_c + \mu & 0 & 0 & 0 \\ 0 & \gamma+\mu & 0 & 0 \\ 0 & 0 & \delta & -\chi \\ 0 & 0 & 0 & \chi \end{pmatrix}
$$

Next we calculate the inverse of the matrix Σ. For our case this turns out to be,

$$
\Sigma^{-1} = \begin{pmatrix} \frac{1}{\gamma\theta(1-\lambda)-\mu_c-\gamma-\mu} & 0 & 0 & 0 \\ 0 & \frac{1}{\gamma+\mu} & 0 & 0 \\ 0 & 0 & \frac{1}{\delta} & \frac{1}{\delta} \\ 0 & 0 & 0 & \frac{1}{\chi} \end{pmatrix}
$$

We then calculate the matrix product $-T \cdot \Sigma^{-1}$.

$$
-T \cdot \Sigma^{-1} = \begin{pmatrix} \frac{\pi\eta N(1-p)(\mu+\epsilon)}{\mu(\mu+\tau+\epsilon)(\gamma\theta(\lambda-1)+\mu_c+\gamma+\mu)} & \frac{\pi\eta N(1-p)(\mu+\epsilon)}{\mu(\gamma+\mu)(\mu+\tau+\epsilon)} & \frac{\pi\alpha N(1-p)(\mu+\epsilon)}{\delta\mu\kappa_L(\mu+\tau+\epsilon)} & \frac{\pi\alpha N(1-p)(\mu+\epsilon)(\chi\kappa_H+\delta\kappa_L)}{\delta\mu\chi\kappa_H\kappa_L(\mu+\tau+\epsilon)} \\ \frac{\pi\eta Np(\mu+\epsilon)}{\mu(\mu+\tau+\epsilon)(\gamma\theta(\lambda-1)+\mu_c+\gamma+\mu)} & \frac{\pi\eta Np(\mu+\epsilon)}{\mu(\gamma+\mu)(\mu+\tau+\epsilon)} & \frac{\pi\alpha Np(\mu+\epsilon)}{\delta\mu\kappa_L(\mu+\tau+\epsilon)} & \frac{\pi\alpha Np(\mu+\epsilon)(\chi\kappa_H+\delta\kappa_L)}{\delta\kappa\mathrm{H}\kappa\mathrm{L}\mu\chi(\mu+\tau+\epsilon)} \\ 0 & 0 & 0 & 0 \\ \frac{(\theta\psi+1-\theta)\xi_S}{W(\gamma\theta(\lambda-1)+\mu_c+\gamma+\mu)} & \frac{\xi_A}{W(\gamma+\mu)} & 0 & 0 \end{pmatrix}
$$

Due to its analytic complexity, an analytic expression for R_0 was not explicitly determined. Instead the characteristic polynomial of $-T \cdot \Sigma^{-1}$ from which the maximum eigenvalue of this expression, was numerically determined.

Jacobian ODE system analysis & related values

$$\begin{pmatrix} -\frac{I\gamma(W_H+\mu)+\gamma\mu(I+N)+(W_H+\mu)(I+N)(R(\epsilon+\kappa_L)+S+\alpha)}{(W_H+\mu)(I+N)} & -R\theta & -R\theta & \mu_C & \tau & -\frac{W_H\gamma\theta}{(W_H+\mu)^2} & -\frac{N\gamma\theta}{(I+N)^2} \\ \frac{(A-1)(-I\gamma(W_H+\mu)+R(W_H+\mu)(I+N)(\epsilon+\kappa_L)(W_L\kappa_H\xi_A+\alpha-\kappa_H(\xi_A-1)+\phi)-\gamma\mu(I+N))}{(W_H+\mu)(I+N)} & R\theta\left(A-1\right)\left(W_L\kappa_H\xi_A+\alpha-\kappa_H\left(\xi_A-1\right)+\phi\right) & R\theta\left(A-1\right)\left(W_L\kappa_H\xi_A+\alpha-\kappa_H\left(\xi_A-1\right)+\phi\right) & 0 & 0 & -\frac{W_H\gamma\theta(A-1)}{(W_H+\mu)^2} & -\frac{N\gamma\theta(A-1)}{(I+N)^2} \\ \frac{A(I\gamma(W_H+\mu)+R(W_H+\mu)(I+N)(\epsilon+\kappa_L)+\gamma\mu(I+N))}{(W_H+\mu)(I+N)} & AR\theta & AR\theta-\alpha-\kappa_H & 0 & 0 & \frac{AW_H\gamma\theta}{(W_H+\mu)^2} & \frac{AN\gamma\theta}{(I+N)^2} \\ 0 & \kappa_H\left(W_L\xi_A-\xi_A+1\right) & \kappa_H & -\alpha-\mu_C & 0 & 0 & 0 \\ S & 0 & 0 & 0 & -\alpha-\tau & 0 & 0 \\ 0 & \frac{\omega(\xi_A\xi_S-\xi_A+1)}{\pi} & \frac{W}{\pi} & 0 & 0 & -V & 0 \\ 0 & 0 & 0 & 0 & 0 & V & -\delta \end{pmatrix}$$

Here we present both a symbolic and numeric form of the Jacobian Matrix of the *V. cholerea* ODE system, the numeric form being at the disease-free equilibrium. The eigenvalues for the disease-free Jacobian are predominantly non-positive but include 2 positive values.

$$\begin{pmatrix} -0.25004 & -0.35399 & -2.7317 & 0.00342 & 0.00137 & 0.0 & 0.0 \\ 0.0 & 0.08194 & 0.0 & 0.0 & 0.0 & 0.0 & 0.0 \\ 0.0 & 0.0 & 2.53166 & 0.0 & 0.0 & 0.0 & 0.0 \\ 0.0 & 0.226 & 0.2 & -0.00347 & 0.0 & 0.0 & 0.0 \\ 0.25 & 0.0 & 0.0 & 0.0 & -0.00141 & 0.0 & 0.0 \\ 0.0 & 16501.33333 & 17.33333 & 0.0 & 0.0 & -1.0 & 0.0 \\ 0.0 & 0.0 & 0.0 & 0.0 & 0.0 & 1.0 & -0.03333 \end{pmatrix}$$

Above we present the numeric form of the DFE Jacobian Matrix. This was calculated using the nominal model parameters applied throughout the simulation. Below are the eigenvalues corresponding to this matrix.

$$-0.0035, \quad -0.2514, \quad 0.0, \quad -0.0333, \quad -1.0, \quad 2.5317, \quad 0.082$$

As with any set of ordinary differential equations, the Jacobian Matrix represents a linear approximation of the flow of the system in phase space at the selected point. The eigenvectors give the direction of flow and the sign of the associated eigenvalues indicates whether the flow is toward or away from the selected point. Given that the Jacobian Matrix is computed at the disease-free equilibrium and there exists positive eigenvalues, we can say that the DFE is an unstable equilibrium (given the nominal chosen model parameters) and the system tends not to equilibriate to the DFE point in phase space [2, 3]. This is consistent with an R_0 value greater than one.

References

1. Diekmann O, Heesterbeek JAP, Roberts MG. The construction of next-generation matrices for compartmental epidemic models. Journal of the Royal Society Interface. 2010 Nov;7(1):873–885.

2. Arrowsmith D, Place CM. Dynamical Systems: Differential Equations, Maps, and Chaotic Behaviour Springer; 1992.

3. Morris H, Stephan S. Differential Equations, Dynamical Systems, and Linear Algebra. Academic Press, Inc; 1974.
